# Clinical characteristics and prognosis of patients with HBV-ACLF versus ALD-ACLF: a retrospective comparative study

**DOI:** 10.3389/fmed.2026.1565646

**Published:** 2026-02-05

**Authors:** Qiuyan Yao, Bohua Tang, Xiaoling Huang, Min Xiao

**Affiliations:** 1 Department of Gastroenterology, The First Affiliated Hospital of Dali University, Dali, Yunnan, China; 2 Department of General Surgery Ward 3, Dali Bai Autonomous Prefecture People’s Hospital, Dali, Yunnan, China; 3 Department of Respiratory, Sichuan Provincial People's Hospital East Sichuan Hospital & Dazhou First People's Hospital, Dazhou, Sichuan, China; 4 Department of Laboratory Medicine, Sichuan Provincial People's Hospital East Sichuan Hospital & Dazhou First People's Hospital, Dazhou, Sichuan, China

**Keywords:** 90-day mortality, acute-on-chronic liver failure, alcohol-associated, clinical characteristics, hepatitis B virus-related

## Abstract

**Objective:**

This study aims to investigate the differences in clinical characteristics and prognosis between patients with HBV-ACLF (Hepatitis B Virus-related Acute-on-Chronic Liver Failure) and those with ALD-ACLF (Alcohol-Associated Acute-on-Chronic Liver Failure), and to identify risk factors associated with 90-day mortality in both cohorts.

**Methods:**

This study enrolled 56 patients with HBV-ACLF and 83 patients with ALD-ACLF to compare their clinical characteristics and conduct analyses of risk factors associated with 90-day prognosis.

**Result:**

Compared with the HBV-ACLF cohort, the ALD-ACLF group exhibited a higher proportion of male patients and a greater prevalence of ascites. Additionally, significant differences were observed in laboratory parameters, with ALD-ACLF patients showing higher levels of WBC (white blood cells), N (neutrophils), and M (monocytes) but lower levels of ALT (alanine aminotransferase), AST (aspartate aminotransferase), ALB (albumin), K (potassium), and Na (sodium) compared to HBV-ACLF patients (*p* < 0.05). In terms of prognostic factors, TBIL (total bilirubin) and PT (prothrombin time) were identified as independent risk factors for 90-day mortality in HBV-ACLF patients, while WBC, TBIL, and PT were associated with 90-day mortality in ALD-ACLF patients.

**Conclusion:**

Patients with ALD-ACLF typically present with a higher prevalence of comorbidities, such as ascites and infections, compared to those with HBV-ACLF. However, no significant differences in prognosis were observed between the two cohorts. For HBV-ACLF patients, elevated TBIL and prolonged PT were identified as independent risk factors for 90-day mortality. In contrast, in addition to TBIL and PT, elevated white WBC was also associated with 90-day mortality in ALD-ACLF patients. These findings warrant further validation through multicenter studies with larger sample sizes.

## 1 Introduction

Acute-on-Chronic Liver Failure (ACLF) is a complex clinical syndrome defined by the acute deterioration of liver function on a background of chronic liver disease, characterized primarily by liver or extrahepatic organ failure (1, 2). ACLF is characterized by complex etiology, rapid progression, and extremely poor prognosis. Despite intensive medical management, mortality remains high, with short-term mortality rates reaching 50–90%, posing a severe threat to human health (3, 4). Thus, early accurate diagnosis and timely, effective treatment are crucial for reducing mortality and improving outcomes in patients with ACLF.

Liver transplantation is the only effective treatment for patients with ACLF. However, its implementation is limited by several factors, including the shortage of donor organs, variable quality of available livers, risks of immune rejection, and the high costs associated with the procedure (3, 5, 6). Given the limited availability of donor livers, many patients are unable to receive timely liver transplantation. Therefore, the current management of ACLF primarily relies on comprehensive medical therapy, which includes general supportive care, hepatoprotective treatments, etiology-specific interventions, and management of complication (5). Among these treatments, etiology-specific therapy for ACLF is particularly crucial. In the Asia-Pacific region and Africa, chronic liver disease is predominantly caused by infection with the hepatitis B virus (HBV). The primary trigger for HBV-related ACLF is the reactivation of HBV-DNA due to discontinuation of antiviral therapy or lack of antiviral treatment (4). In Europe and North America, chronic liver disease is predominantly characterized by alcohol-related cirrhosis, with excessive alcohol consumption, alcoholic hepatitis, and bacterial infections being the primary precipitating factors for alcohol-associated ACLF (7, 8).

Although alcohol-related acute-on-chronic liver failure (ALD-ACLF) and hepatitis B virus-related acute-on-chronic liver failure (HBV-ACLF) share some clinical similarities, such as jaundice, ascites, hepatomegaly, and coagulopathy, their underlying causes and precipitating factors differ significantly (9). Despite sharing some clinical similarities, alcohol-related ACLF (ALD-ACLF) and hepatitis B virus-related ACLF (HBV-ACLF) exhibit significant differences in etiology, pathogenesis, clinical features, laboratory findings, and therapeutic approaches. These differences dictate distinct clinical management and treatment strategies. Targeted treatment and management of these two ACLF subtypes are crucial for improving patient outcomes. For HBV-ACLF patients with positive HBV-DNA, rapid reduction of HBV-DNA levels at an early stage is the key to effective therapy (10). For patients with alcohol-related acute-on-chronic liver failure (ALD-ACLF), abstinence from alcohol is of paramount importance. International guidelines recommend using the Model for End-Stage Liver Disease (MELD) score to assess the need for corticosteroid therapy. However, this approach may not be universally applicable to the Chinese population. In cases where patients have complications such as infections, the use of corticosteroids requires careful consideration.

Although previous studies have explored the differences between acute-on-chronic liver failure (ACLF) caused by hepatitis B virus (HBV) and alcohol-related liver disease, a comprehensive comparison of these two types of ACLF remains insufficient. This study aims to analyze the clinical characteristics and outcomes of patients with alcohol-related ACLF and HBV-related ACLF, and to identify risk factors for 90-day mortality. By comparing clinical data from both cohorts, we aim to reveal differences in clinical presentation, laboratory findings, and prognosis. This comparison will enhance our understanding of the complexity of ACLF caused by these two etiologies and provide a basis for clinicians to develop effective interventions. Identifying key factors influencing outcomes will facilitate the development of individualized treatment plans, thereby improving patient survival rates and quality of life.

## 2 Materials and methods

### 2.1 General information

**Figure 1:**
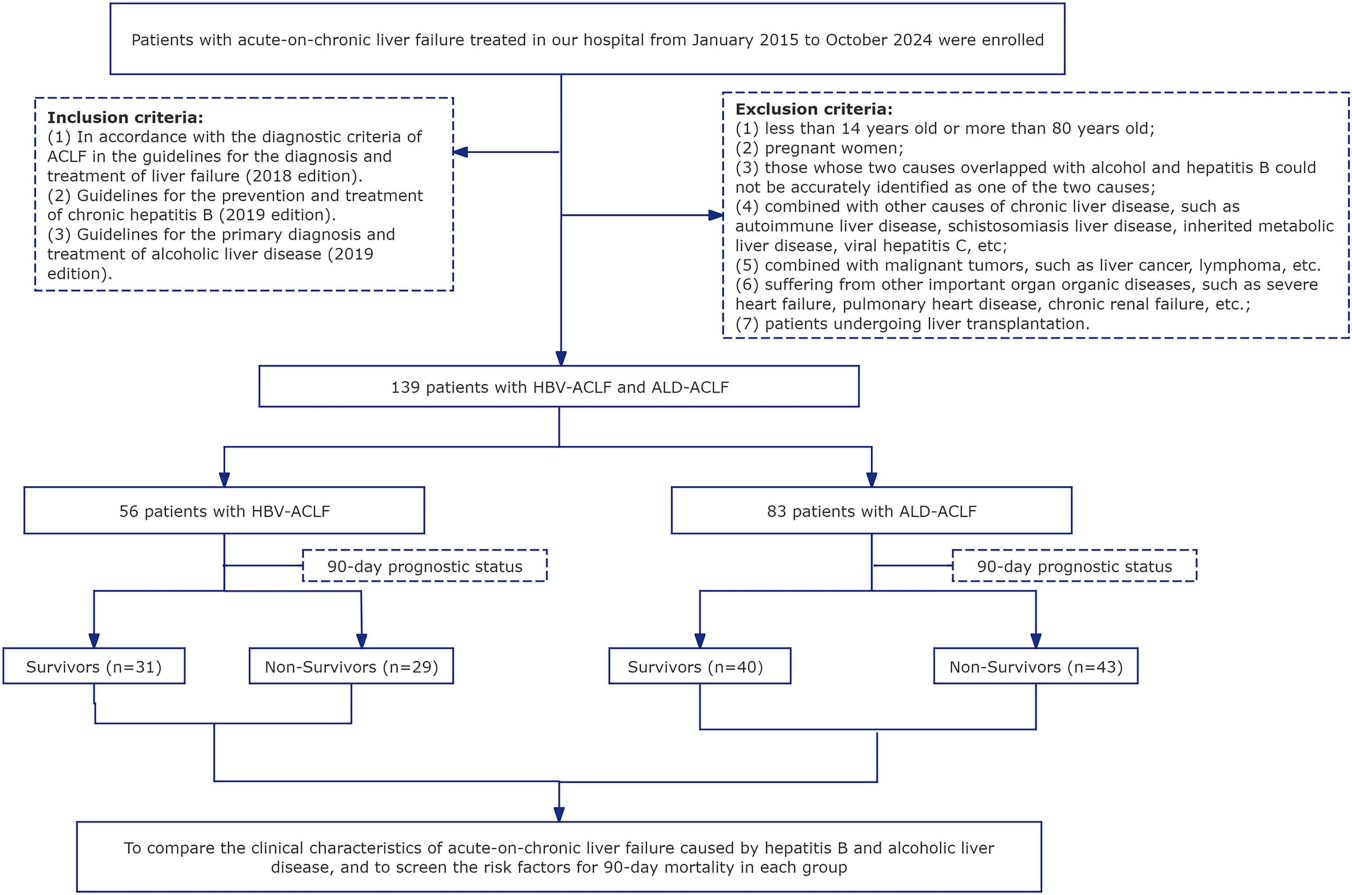
Flow chart of the study.

In this study, we enrolled 56 patients with HBV-ACLF and 83 patients with ALD-ACLF from the First Affiliated Hospital of Dali University, spanning the period from January 2015 to October 2024 (Figure 1). This study spans nearly a decade, during which significant advancements have been made in diagnostic criteria and treatment modalities (e.g., artificial liver support, antiviral regimens, and ICU management). However, as our hospital is located in a remote region of China, these advanced technologies only became available in recent years due to earlier resource limitations. We acknowledge that evolving diagnostic standards and treatment protocols during this extended period may have influenced our findings, although these temporal variations were not specifically accounted for in our study design. This represents an important limitation of our research.

Patients included in this study were required to meet the following diagnostic criteria: 1. ACLF Diagnostic Criteria: All patients met the diagnostic criteria for ACLF as defined in the Guidelines for the Diagnosis and Treatment of Liver Failure (10). 2 Chronic Hepatitis B Diagnostic Criteria: Based on the Guidelines for the Prevention and Treatment of Chronic Hepatitis B (11), HBV infection was defined as positivity for HBsAg and/or HBV DNA. Chronic infection was defined as positivity for HBsAg and/or HBV DNA for more than 6 months. 3. Alcoholic Liver Disease Diagnostic Criteria: Based on the Primary Care Guidelines for the Diagnosis and Treatment of Alcoholic Liver Disease (12): (1) Long-term Alcohol Consumption History: Generally more than 5 years, with an ethanol intake of ≥40 g/d for males or ≥20 g/d for females, or a history of heavy alcohol consumption within 2 weeks, with an ethanol intake of >80 g/d. The ethanol content (g) was calculated using the formula: ethanol content (g) = alcohol volume (mL) × ethanol percentage × 0.8. (2) Laboratory Abnormalities: Elevated levels of alanine aminotransferase (ALT), aspartate aminotransferase (AST), *γ*-glutamyl transpeptidase (GGT), total bilirubin (TBIL), prothrombin time (PT), and mean red blood cell volume (MCV), with AST/ALT ratio typically >2. (3) Clinical Manifestations: Presence of clinical symptoms and signs. (4) Imaging Findings: Typical radiological features consistent with alcoholic liver disease. 4. Cirrhosis Diagnostic Criteria: (1) Definite Etiology: A clear underlying cause of liver disease. (2) Histopathological Confirmation: Liver biopsy showing histological features consistent with cirrhosis. (3) Clinical Diagnosis: Meeting at least two of the following five criteria, excluding non-cirrhotic portal hypertension: 1) Imaging findings suggestive of cirrhosis and/or portal hypertension. 2) Endoscopic evidence of esophageal or gastric varices. 3) Liver stiffness measurements consistent with cirrhosis. 4) Biochemical evidence of hypoalbuminemia (<35 g/L) and/or prolonged PT (>3 s compared to controls). 5) Hematological evidence of thrombocytopenia (<100 × 10^9/L). A diagnosis of cirrhosis was considered histopathological if criteria (1) and (2) were met, or clinical if criteria (1) and (3) were met.

Exclusion criteria: 1. Age less than 14 years or greater than 80 years. 2. Pregnant or lactating individuals. 3. Patients with overlapping etiologies of alcohol and hepatitis B, making it impossible to accurately attribute the cause to one or the other. 4. Presence of other etiologies leading to chronic liver disease, such as autoimmune hepatitis, schistosomal liver disease, hereditary metabolic liver disease, or hepatitis C virus infection. 5. Patients with malignancies, such as hepatocellular carcinoma or lymphoma. 6. Presence of other significant organ diseases: such as severe heart failure, cor pulmonale, or chronic renal failure. 7. Patients who have undergone liver transplantation.

All enrolled patients were treated at our regional tertiary hospital in accordance with the relevant international and domestic guidelines at the time. For ALD-ACLF patients, abstinence from alcohol and nutritional support form the cornerstone of treatment. Corticosteroids were considered for those with severe alcoholic hepatitis who met specific criteria (e.g., MDF > 32 and absence of contraindications such as active infection). For patients complicated by hepatorenal syndrome or severe electrolyte disturbances, CRRT was initiated.

Regarding the Relationship Between Disease Severity and Treatment Intensity: To indirectly assess whether the intensity of treatment influenced comparability between the groups, we further analyzed indicators reflecting disease severity and inflammatory status. As shown in Table 1, although the ALD-ACLF group had higher infection rates and inflammatory markers (WBC, N), there were no statistically significant differences between the two groups in baseline MELD score, Child-Pugh score, or 90-day survival rate. This suggests that, despite differing etiologies, the overall disease severity and short-term prognosis risk at the time of enrollment were similar between the groups. Therefore, the intensity of comprehensive treatment received may have been comparable at the population level.

**Table 1:** Comparison of characteristics between the HBV-ACLF and ALD-ACLF groups.

Variables	Total (*n* = 139)	HBV-ACLF (*n* = 56)	ALD-ACL (*n* = 83)	*p*-value
Patient characteristics				
Age	51 (41, 57)	51 (40, 56)	51 (43, 57)	0.757
Sex				<0.001
Male	114 (82.0%)	38 (67.9%)	76 (91.6%)	
Female	25 (18.0%)	18 (32.1%)	7 (8.4%)	
Comorbidities				
Hydroperitoneum				<0.001
No	17 (12.2%)	14 (25.0%)	3 (3.6%)	
Yes	122 (87.8%)	42 (75.0%)	80 (96.4%)	
Infections				0.042
No	39 (28.1%)	21 (37.5%)	18 (21.7%)	
Yes	100 (71.9%)	35 (62.5%)	65 (78.3%)	
Hemorrhage				0.977
No	119 (85.6%)	48 (85.7%)	71 (85.5%)	
Yes	20 (14.4%)	8 (14.3%)	12 (14.5%)	
Hepatic encephalopathy				0.683
No	117 (84.2%)	48 (85.7%)	69 (83.1%)	
Yes	22 (15.8%)	8 (14.3%)	14 (16.9%)	
Laboratory indices (median, IQR)				
White blood cell (×10^9^/L)	9 (6, 14)	7 (6, 9)	11 (7, 17)	<0.001
Neutrophil (×10^9^/L)	6 (4, 11)	5 (4, 6)	9 (5, 13)	<0.001
Lymphocyte (×10^9^/L)	1.18 (0.83, 1.79)	1.15 (0.81, 1.76)	1.23 (0.84, 1.79)	0.717
Monocytes (×10^9^/L)	0.88 (0.61, 1.24)	0.69 (0.49, 1.00)	0.96 (0.70, 1.34)	<0.001
Platelet (×10^9^/L)	99 (60, 139)	98 (61, 124)	100 (59, 143)	0.569
Total bilirubin (umol/L)	248 (193, 354)	252 (212, 339)	238 (190, 357)	0.643
Alanine aminotransferase	55 (31, 142)	185 (83, 529)	36 (25, 57)	<0.001
Aspartate aminotransferase	116 (86, 239)	229 (112, 506)	94 (75, 149)	<0.001
Albumin (g/L)	26.8 (22.8, 29.5)	28.7 (24.6, 32.0)	25.3 (22.1, 28.5)	0.001
Creatinine	71 (54, 91)	71 (54, 88)	71 (52, 95)	0.879
Blood urea nitrogen (mmol/L)	5.0 (3.5, 7.5)	4.9 (3.6, 6.8)	5.3 (3.5, 7.9)	0.686
Potassium (mmol/L)	3.70 (3.18, 4.16)	3.95 (3.47, 4.30)	3.58 (3.11, 3.91)	0.002
Sodium (mmol/L)	136.0 (133.5, 140.0)	138.0 (134.0, 141.0)	136.0 (132.5, 139.0)	0.016
Prothrombin time (s)	21.7 (18.7, 24.9)	21.8 (18.7, 26.4)	21.7 (18.8, 24.5)	0.532
International standardized ratio	1.93 (1.60, 2.30)	1.93 (1.60, 2.45)	1.93 (1.61, 2.21)	0.522
Fibrinogen (g/L)	1.75 (1.27, 2.29)	1.54 (1.23, 1.99)	1.82 (1.34, 2.49)	0.051
Disease severity (median, IQR)				
MELD score	25.0 (22.0, 28.0)	25.0 (22.0, 28.0)	24.0 (22.0, 28.0)	0.616
Child-Pugh score	12.00 (11.00, 13.00)	12.00 (10.75, 13.00)	12.00 (11.00, 13.00)	0.097
Outcomes (*n*, %)				
Survivors				0.678
No	70 (50.4%)	27 (48.2%)	43 (51.8%)	
Yes	69 (49.6%)	29 (51.8%)	40 (48.2%)	

### 2.2 Detection index and method

Upon admission, basic information was collected from patients, including age, sex, underlying liver disease status (cirrhosis/non-cirrhosis), and grouping based on 90-day survival status. At 6 a.m., fasting venous blood samples were obtained from patients for routine blood tests, coagulation function, and biochemical analyses.

Scoring calculations were as follows: MELD score = 9.6 × ln [SCr (mg/dL)] + 3.8 × ln [TBIL (mg/dL)] + 11.2 × ln (INR) + 6.4 × etiology (biliary or alcoholic = 0; other etiologies = 1). Maddrey Discriminant Function (MDF) = 4.6 × (patient PT – control PT) (s) + TBIL (mg/dL) (Note: MDF was calculated only for patients in the ALD-ACLF group).

### 2.3 Statistical analysis

Data were processed using the R programming language and SPSS version 26.0. For quantitative data that followed a normal distribution, intergroup comparisons were performed using the independent samples *t*-test. Quantitative data that did not follow a normal distribution were presented as medians with interquartile ranges M(Q1, Q3), and intergroup comparisons were conducted using the Mann–Whitney U test. Categorical data were expressed as counts and percentages n(%) and intergroup comparisons were performed using the χ^2^ test. Initially, baseline clinical characteristics were compared between patients with HBV-ACLF and those with ALD-ACLF. Subsequently, risk factors for 90-day mortality were identified in each group and subjected to collinearity analysis to exclude collinear variables. After excluding collinear variables, the optimal cutoff values of PLT, TBIL, PT, ALB, Na, and WBC were determined by receiver operating characteristic (ROC) curve analysis for classification. Values below the cutoff were assigned to the low-level group, while values equal to or above the cutoff were assigned to the high-level group. Univariate and multivariate logistic regression analyses were then conducted to identify independent risk factors for 90-day mortality in patients with HBV-ACLF and ALD-ACLF, respectively. Variables with *p* < 0.05 in the multivariate logistic regression analysis of 90-day mortality were included in the nomogram construction. Model performance was validated using ROC curves and calibration curves, and clinical utility was assessed using decision curve analysis (DCA).

## 3 Results

### 3.1 Comparison of clinical characteristics between the HBV-ACLF and ALD-ACLF groups

**Figure 2:**
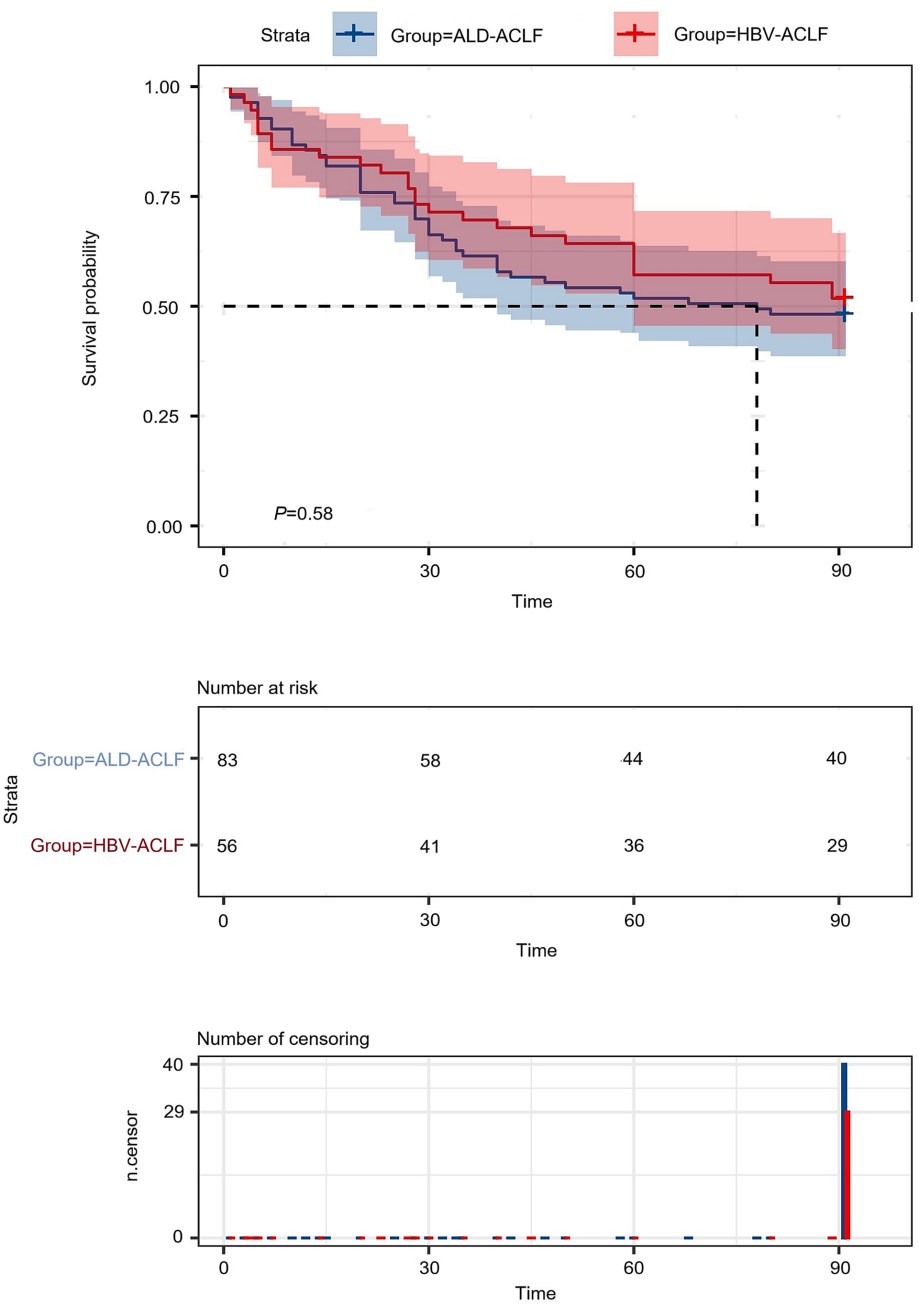
Survival curves of HBV-ACLF patients versus ALD-ACLF patients.

A total of 56 patients with HBV-ACLF and 83 patients with ALD-ACLF were included. No significant differences were observed between the two groups in terms of bleeding, hepatic encephalopathy, or levels of L, PLT, TBIL, Cr, BUN, PT, INR, FIB, MELD score, Child-Pugh score or survival rate (*p* > 0.05). The survival curve indicates no statistically significant difference in 90-day mortality between the hepatitis B-related acute-on-chronic liver failure group and the alcohol-related acute-on-chronic liver failure group (Figure 2). However, compared with the HBV-ACLF group, the ALD-ACLF group had a higher proportion of male patients and a greater prevalence of infections and ascites. Additionally, the ALD-ACLF group exhibited significantly higher levels of WBC, N, and M, but lower levels of ALT, AST, ALB, K, and Na (*p* < 0.05) (Table 1). Liver Transplantation Status It is a critical contextual point that none of the 139enrolled patients (0%) underwent liver transplantation during the index hospitalization for ACLF or within the 90-day follow-up period. This reflects the real-world constraints of our regional medical center during the study timeframe and ensures that the subsequent survival and prognostic analyses pertain exclusively to outcomes under comprehensive medical management.

### 3.2 Risk factors analysis for 90-day mortality in the HBV-ACLF group

Patients with HBV-ACLF were stratified into survival and non-survival groups based on 90-day outcomes. Compared with the survival group, the non-survival group exhibited greater age, higher prevalence of bleeding and hepatic encephalopathy, elevated TBIL, prolonged PT, INR, and higher MELD and Child-Pugh scores. Additionally, the non-survival group had significantly lower levels of PLT, ALB, and Na (Table 2). To assess multicollinearity among these variables, variance inflation factor (VIF) analysis was conducted, with VIF values greater than 5 indicating significant collinearity. After excluding collinear variables, the retained indicators (PLT, TBIL, ALB, Na, and PT) all had VIF values below 5, confirming the absence of multicollinearity.

**Table 2:** Comparison of characteristics between the 90-day survival and non-survival groups.

Variables	HBV-ACLF (*n* = 56)			ALD-ACL (*n* = 83)		
	Survivors (*n* = 31)	Non-survivors (*n* = 29)	*P*-value	Survivors (*n* = 40)	Non-survivors (*n* = 43)	*P*-value
Patient characteristics						
Age	46 (37, 53)	54 (45, 62)	0.003	51 (44, 58)	47 (43, 56)	0.544
Sex			0.698			0.706
Male	19 (65.5%)	19 (70.4%)		36 (90.0%)	40 (93.0%)	
Female	10 (34.5%)	8 (29.6%)		4 (10.0%)	3 (7.0%)	
Comorbidities						
Hydroperitoneum			0.877			0.108
No	7 (24.1%)	7 (25.9%)		3 (7.5%)	0 (0.0%)	
Yes	22 (75.9%)	20 (74.1%)		37 (92.5%)	43 (100.0%)	
Infections			0.945			0.021
No	11 (37.9%)	10 (37.0%)		13 (32.5%)	5 (11.6%)	
Yes	18 (62.1%)	17 (63.0%)		27 (67.5%)	38 (88.4%)	
Hemorrhage			0.023			0.892
No	28 (96.6%)	20 (74.1%)		34 (85.0%)	37 (86.0%)	
Yes	1 (3.4%)	7 (25.9%)		6 (15.0%)	6 (14.0%)	
Hepatic encephalopathy			0.023			0.661
No	28 (96.6%)	20 (74.1%)		34 (85.0%)	35 (81.4%)	
Yes	1 (3.4%)	7 (25.9%)		6 (15.0%)	8 (18.6%)	
Laboratory indices (median, IQR)						
White blood cell (×10^9^/L)	6.6 (5.4, 9.2)	7.2 (5.8, 9.2)	0.884	10 (7, 13)	14 (9, 19)	0.010
Neutrophil (×10^9^/L)	4.6 (3.3, 5.9)	4.6 (4.0, 6.5)	0.560	7 (4, 9)	11 (7, 17)	0.010
Lymphocyte (×10^9^/L)	1.37 (0.93, 1.74)	1.04 (0.77, 1.66)	0.173	1.25 (0.96, 1.78)	1.22 (0.77, 1.83)	0.610
Monocytes (×10^9^/L)	0.68 (0.50, 0.87)	0.72 (0.49, 1.08)	0.682	0.95 (0.63, 1.19)	1.14 (0.78, 1.52)	0.074
Platelet (×109/L)	118 (88, 146)	81 (47, 113)	0.004	95 (50, 141)	100 (69, 144)	0.417
Total bilirubin (umol/L)	221 (192, 271)	335 (240, 441)	0.002	192 (170, 250)	339 (233, 454)	<0.001
Alanine aminotransferase	333 (118, 965)	122 (82, 438)	0.123	39 (25, 55)	34 (26, 60)	0.813
Aspartate aminotransferase	268 (146, 538)	173 (106, 365)	0.098	90 (78, 126)	99 (72, 153)	0.512
Albumin (g/L)	30.6 (25.6, 33.3)	26.9 (22.4, 29.0)	0.006	26.1 (22.4, 28.8)	24.5 (22.1, 28.3)	0.121
Creatinine	69 (51, 77)	73 (56, 89)	0.436	64 (51, 83)	76 (58, 106)	0.065
Blood urea nitrogen (mmol/L)	4.7 (3.5, 6.0)	5.4 (3.7, 9.5)	0.150	4.8 (3.4, 7.3)	6.0 (3.6, 9.5)	0.312
Potassium (mmol/L)	3.93 (3.49, 4.30)	3.96 (3.47, 4.31)	0.974	3.62 (3.12, 3.91)	3.54 (3.02, 3.91)	0.884
Sodium (mmol/L)	140.0 (137.0, 141.0)	135.0 (133.0, 139.5)	0.048	136.0 (134.0, 138.0)	135.0 (132.0, 139.0)	0.667
Prothrombin time (s)	18.9 (17.9, 21.7)	25.3 (22.9, 28.0)	<0.001	19.7 (18.0, 21.8)	24.2 (20.7, 27.1)	<0.001
International standardized ratio	1.66 (1.53, 1.91)	2.36 (2.03, 2.66)	<0.001	1.70 (1.56, 1.95)	2.17 (1.82, 2.53)	<0.001
Fibrinogen (g/L)	1.80 (1.39, 2.02)	1.38 (1.10, 1.83)	0.112	1.95 (1.51, 2.49)	1.69 (1.15, 2.48)	0.257
Disease severity (median, IQR)						
MELD score	22.0 (21.0, 25.0)	28.0 (25.5, 31.5)	<0.001	23.0 (21.0, 24.0)	27.0 (25.0, 30.0)	<0.001
Child-Pugh score	11.00 (10.00, 12.00)	13.00 (11.50, 13.00)	<0.001	11.50 (11.00, 12.25)	13.00 (12.00, 13.00)	<0.001

**Figure 3:**
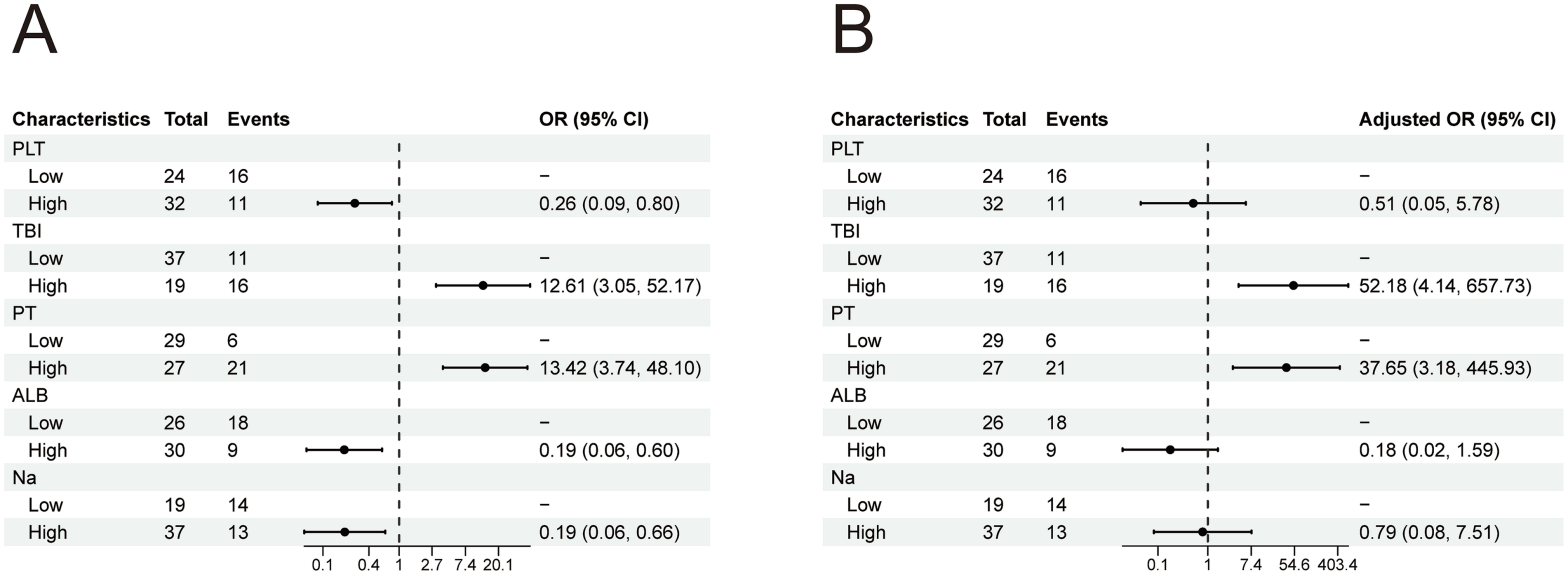
**(A)** Univariate logistic analysis of patients in the HBV-ACLF group. **(B)** Multivariate logistic analysis of patients in the HBV-ACLF group.

Given that the aforementioned differential indicators did not follow a normal distribution, receiver operating characteristic (ROC) curve analysis was employed to determine the cutoff values for these parameters (Table 3). The cutoff values were as follows: 92.0 for PLT, 317.3 for TBIL, 28.4 for ALB, 136.0 for Na, and 22.7 for PT. After dichotomizing these indicators based on the cutoff values, univariate and multivariate logistic regression analyses were performed to assess risk factors associated with 90-day mortality in HBV-ACLF patients (Table 4). Subsequently, multivariate logistic regression revealed that elevated TBIL and prolonged PT were independent risk factors for 90-day mortality in HBV-ACLF patients (Figure 3).

**Table 3:** ROC analysis of prognostic indicators for the HBV-ACLF and ALD-ACLF groups.

Predictor	AUC (95% CI)	Cut Off-value	Youden Index J	Sensitivity	Specificity
HBV-ACLF					
Platelet (×10^9^/L)	0.727 (0.593–0.861)	92.0	0.39	66.7%	72.4%
Total bilirubin (umol/L)	0.746 (0.610–0.882)	317.3	0.49	59.3%	89.7%
Albumin (g/L)	0.716 (0.578–0.855)	28.4	0.43	70.4%	72.4%
Sodium (mmol/L)	0.655 (0.504–0.805)	136.0	0.35	55.6%	79.3%
Prothrombin Time (s)	0.814 (0.696–0.933)	22.7	0.57	77.8%	79.3%
ALD-ACL					
White blood cell (×10^9^/L)	0.664 (0.545–0.783)	12.83	0.36	77.5%	58.1%
Total bilirubin (umol/L)	0.781 (0.679–0.883)	219.4	0.49	83.7%	65.0%
Prothrombin time (s)	0.790 (0.692–0.888)	22.2	0.50	69.8%	80.0%

**Table 4:** Logistic regression analysis of patients with HBV-ACLF.

Index	Univariable			Multivariable		
	OR	95%CI	*P*-value	OR	95%CI	*P*-value
PLT						
Low (<92.0 × 10^9^/L)	–	–		–	–	
High (≥92.0 × 10^9^/L)	0.26	0.09, 0.80	0.019	0.51	0.05, 5.78	0.587
TBIL						
Low (<317.3 umol/L)	–	–		–	–	
High (≥317.3 umol/L)	12.61	3.05, 52.17	<0.001	52.18	4.14, 657.73	0.002
PT						
Low (<22.7 s)	–	–		–	–	
High (≥22.7 s)	13.42	3.74, 48.10	<0.001	37.65	3.18, 445.93	0.004
ALB						
Low (<28.4 g/L)	–	–		–	–	
High (≥28.4 g/L)	0.19	0.06, 0.60	0.004	0.18	0.02, 1.59	0.122
Na						
Low (<136.0 mmol/L)	–	–		–	–	
High (≥136.0 mmol/L)	0.19	0.06, 0.66	0.009	0.79	0.08, 7.51	0.836

**Figure 4:**
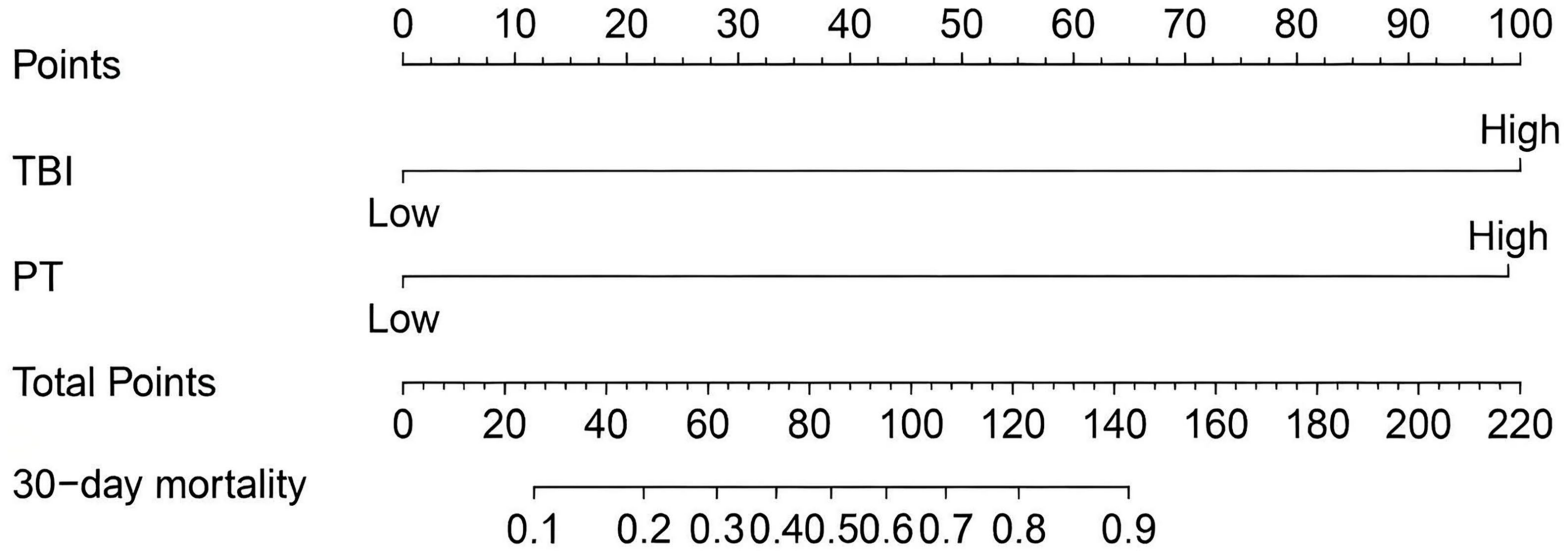
Nomogram for predicting prognosis in patients with HBV-ACLF.

**Figure 5:**
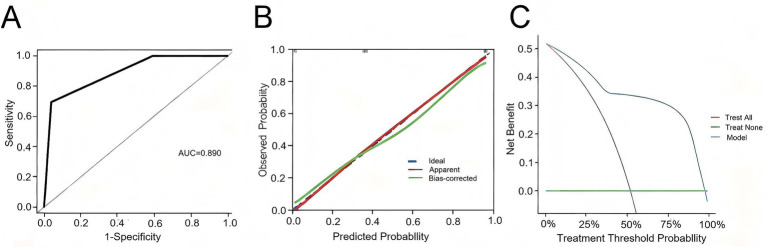
**(A)** ROC curve of the predictive model for HBV-ACLF patients. **(B)** Calibration curve of the predictive model for HBV-ACLF patients. **(C)** Clinical decision curve of the predictive model for HBV-ACLF patients.

A nomogram model was constructed based on the independent risk factors for 90-day mortality in HBV-ACLF patients (Figure 4). The ROC curve analysis revealed an area under the curve (AUC) of 0.890 for this model, indicating robust predictive performance (Figure 5A). Calibration of the model was assessed using calibration curves, with the bias-corrected line closely approximating the ideal line, suggesting good agreement between predicted and actual outcomes (Figure 5B). Decision curve analysis (DCA) was employed to evaluate the clinical utility of the model. The results demonstrated that, compared with strategies of intervening in all or no patients, the nomogram model provided greater net benefit for early prediction and intervention in HBV-ACLF patients (Figure 5C).

### 3.3 Risk factors analysis for 90-day mortality in the ALD-ACLF group

Patients with ALD-ACLF were stratified into survival and non-survival groups based on their 90-day outcomes. Compared with the survival group, the non-survival group exhibited a higher prevalence of infections and elevated levels of WBC, N, and TBIL, as well as prolonged PT and INR. Additionally, both the MELD score and Child-Pugh score were higher in the non-survival group. To assess multicollinearity among these differential indicators, VIF analysis was conducted. A VIF value greater than 5 indicates significant collinearity between variables. After excluding collinear variables, WBC, TBIL, and PT were retained, with all VIF values below 5, confirming the absence of multicollinearity.

**Figure 6:**
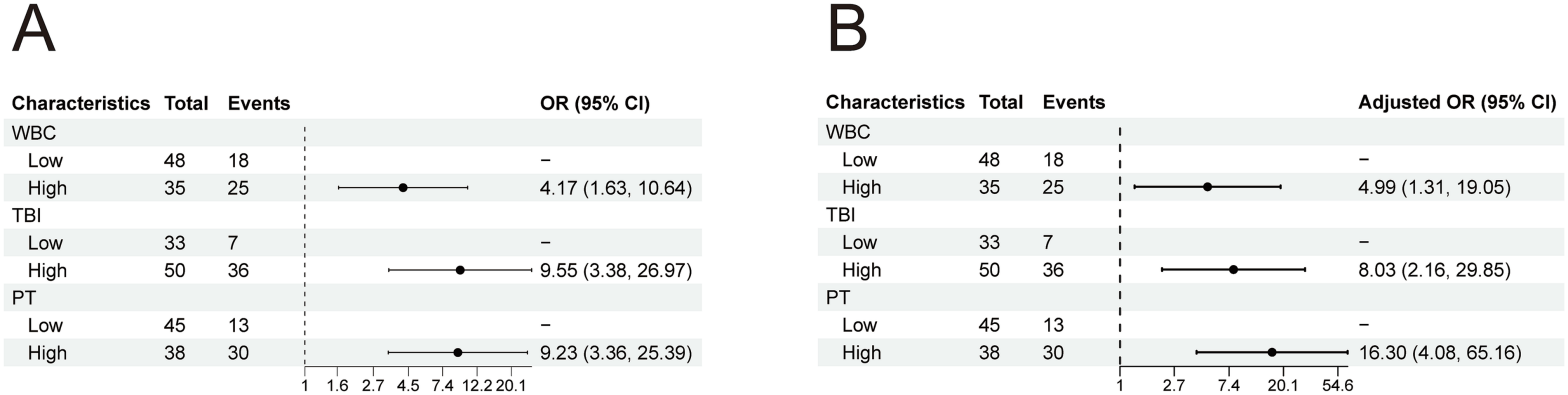
**(A)** Univariate logistic analysis of patients in the ALD-ACLF group. **(B)** Multivariate logistic analysis of patients in the ALD-ACLF group.

Given that the aforementioned differential indicators did not follow a normal distribution, ROC curve analysis was employed to determine the cutoff values for these parameters (Table 3). The cutoff values were as follows: 12.83 for WBC, 219.4 for TBIL, and 22.2 for PT. After dichotomizing these indicators based on the cutoff values, univariate and multivariate logistic regression analyses were performed to assess risk factors associated with 90-day mortality in ALD-ACLF patients (Table 5). Subsequently, multivariate logistic regression revealed that elevated WBC, TBIL, and prolonged PT were independent risk factors for 90-day mortality in ALD-ACLF patients (Figure 6).

**Table 5:** Logistic regression analysis of patients with ALD-ACLF.

Index	Univariable			Multivariable		
	OR	95%CI	*P*-value	OR	95%CI	*P*-value
WBC	–	–		–	–	
Low (<12.83 × 10^9^/L)	4.17	1.63, 10.64	0.003	4.99	1.31, 19.05	0.019
High (≥12.83 × 10^9^/L)						
TBIL	–	–		–	–	
Low (<219.4 umol/L)	9.55	3.38, 26.97	<0.001	8.03	2.16, 29.85	0.002
High (≥219.4 umol/L)						
PT	–	–		–	–	
Low (<22.2 s)	9.23	3.36, 25.39	<0.001	16.30	4.08, 65.16	<0.001
High (≥22.2 s)	–	–		–	–	

**Figure 7:**
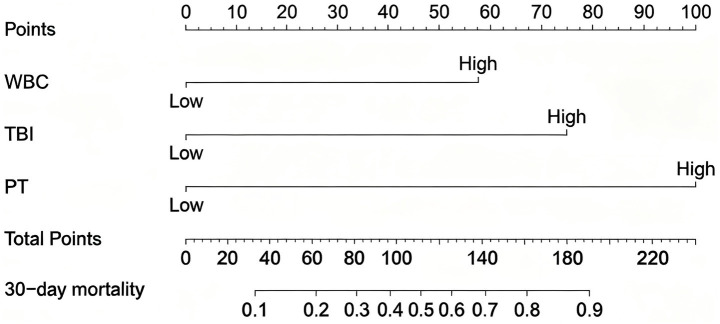
Nomogram for predicting prognosis in patients with ALD-ACLF.

**Figure 8:**
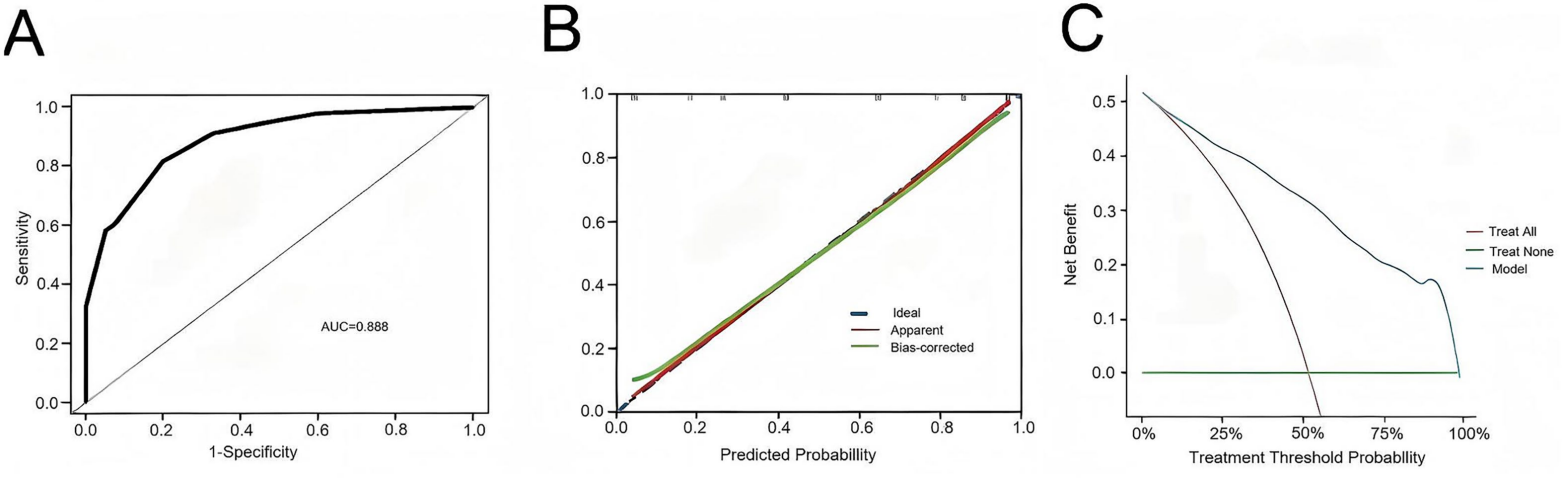
**(A)** ROC curve of the predictive model for ALD-ACLF patients. **(B)** Calibration curve of the predictive model for ALD-ACLF patients. **(C)** Clinical decision curve of the predictive model for ALD-ACLF patients.

A nomogram model was constructed based on the independent risk factors for 90-day mortality in ALD-ACLF patients (Figure 7). The ROC analysis of this model yielded an AUC of 0.888, indicating robust predictive performance (Figure 8A). Calibration of the model was assessed using calibration curves, with the bias-corrected line closely approximating the ideal line, suggesting good agreement between predicted and actual outcomes (Figure 8B). DCA was employed to evaluate the clinical utility of the model. The results demonstrated that, compared with strategies of intervening in all or no patients, the nomogram model provided greater net benefit for early prediction and intervention in ALD-ACLF patients (Figure 8C).

## 4 Discussion

Acute-on-Chronic Liver Failure (ACLF) progresses rapidly and has a poor prognosis, posing a serious threat to human health. Even with comprehensive medical treatment, the short-term mortality rate remains as high as 50–90%. The etiology of ACLF exhibits significant regional variations (3, 4). In the Asia-Pacific region and Africa, hepatitis B virus (HBV) infection is the primary cause, with HBV-DNA reactivation due to drug withdrawal or irregular treatment being the main trigger. In contrast, in Europe and America, alcohol-related liver cirrhosis is the underlying condition, with excessive alcohol consumption, alcoholic hepatitis, and bacterial infections serving as key precipitating factors. These differences may lead to variations in clinical features, disease progression, and prognosis between the two types of ACLF patients (7, 8). In light of this, our study conducted an in-depth comparison of the clinical characteristics and prognosis between HBV-ACLF and ALD-ACLF (alcohol-related liver disease ACLF) patients and identified risk factors for 90-day mortality. The key findings are as follows: (1) The proportion of male patients in the ALD-ACLF group was significantly higher than in the HBV-ACLF group. (2) WBC and neutrophil (Neu) levels were markedly elevated in the ALD-ACLF group compared to the HBV-ACLF group. (3) Patients in the ALD-ACLF group were more prone to complications such as ascites and infections than those in the HBV-ACLF group. (4) TBIL (total bilirubin) and PT (prothrombin time) may be independent risk factors for 90-day mortality in HBV-ACLF patients, whereas TBIL, PT, and WBC were identified as independent risk factors for 90-day mortality in ALD-ACLF patients. This retrospective study aimed to compare the clinical characteristics, prognosis, and risk factors for 90-day. In review mortality between patients with HBV-ACLF and those with ALD-ACLF. Our main findings are as follows: First, compared with HBV-ACLF patients, ALD-ACLF patients had a higher proportion of males, were more prone to ascites and infections, and showed significantly higher levels of inflammatory markers such as white blood cells and neutrophils, while exhibiting lower levels of ALT, AST, and ALB. Second, despite differences in the incidence of complications between the two groups, there were no statistically significant differences in 90-day survival rates or disease severity scores (MELD, Child-Pugh). Finally, multivariate regression analysis identified TBIL and PT as independent risk factors for 90-day mortality in HBV-ACLF patients; for ALD-ACLF patients, in addition to TBIL and PT, elevated WBC was also an independent risk factor.

First, this study found that there were certain differences in the initial clinical characteristics between HBV-ACLF and ALD-ACLF patients. Although both groups were predominantly male, the proportion of male patients in the ALD-ACLF group was significantly higher. This phenomenon is primarily attributed to gender differences in alcohol consumption habits and lifestyle. Compared to women, men are not only more likely to engage in long-term heavy drinking but may also rely more on alcohol to relieve stress due to greater social and work-related pressures. Such unhealthy drinking behavior increases the risk of liver disease, leading to hepatic inflammation, progressive fibrosis, and cirrhosis, which accelerate liver damage and ultimately predispose individuals to ALD-ACLF (7). However, due to the limited sample size of this study, these findings still require further validation through larger-scale research.

The study demonstrated that patients in the ALD-ACLF group exhibited a significantly higher incidence of ascites compared to the HBV-ACLF group (96.4% vs. 75.0%). This disparity may be attributed to the direct effects of alcohol and its metabolites, which can damage vascular endothelial cells and increase vascular permeability, thereby promoting fluid extravasation from blood vessels into surrounding tissues—particularly accumulating in the peritoneal cavity to form ascites (10). Furthermore, impaired hepatic synthetic function leading to hypoalbuminemia and hormonal dysregulation also serve as significant contributing factors to ascites formation.

Systemic inflammatory response serves as a hallmark feature of ACLF, particularly in alcohol-related liver disease where it acts as a key driver for the onset and progression of ACLF (11). Our findings substantiate this perspective through multiple lines of evidence: Clinically, the ALD-ACLF group demonstrated a significantly higher incidence of infection compared to the HBV-ACLF group (78.3% vs. 62.5%, *p* < 0.05). Laboratory parameters revealed markedly elevated white blood cell (WBC) and neutrophil (Neu) counts in ALD-ACLF patients, with deceased patients showing even more pronounced elevations than survivors. Multivariate logistic regression analysis further identified WBC as an independent risk factor for 90-day mortality in ALD-ACLF patients. These collective results indicate that ALD-ACLF patients not only face greater infection risks but also exhibit more severe inflammatory responses, which likely constitute critical determinants of disease progression and clinical outcomes.

Crucially, our data indicate that the infections observed were predominantly present at admission, underscoring their role as potential triggers or concomitant exacerbating factors in the acute decompensation of ALD, rather than mere sequelae of prolonged hospitalization. This finding reinforces the idea that active infection initial clinical presentation and pathogenesis of ALD-ACLF. and the resultant systemic inflammatory response are integral to the initial clinical presentation and pathogenesis of ALD-ACLF. The heightened susceptibility to infections in ALD-ACLF patients may involve multiple mechanisms: (1) Alcohol-induced gut dysbiosis: Alcohol directly disrupts the intestinal microbiota, increasing pathogenic bacteria while reducing beneficial species. This promotes bacterial overgrowth and destabilizes the gut microecological balance. (2) Gut barrier dysfunction: Alcohol exerts direct toxicity on intestinal epithelial cells, downregulating tight junction proteins and increasing mucosal permeability. This facilitates the translocation of gut-derived pathogen-associated molecular patterns (PAMPs) across the intestinal barrier into the liver (12–14). Additionally, alcohol impairs gut-associated lymphoid tissue, increasing activated monocytes, dendritic cells, and lymphocytes. This upregulates proinflammatory cytokines, further damaging epithelial tight junctions and exacerbating bacterial translocation, creating a vicious cycle of “gut injury → inflammation → barrier disruption.” (3) Hepatic oxidative stress and DAMP release: Alcohol and its metabolites induce mitochondrial dysfunction and endoplasmic reticulum stress in hepatocytes, triggering reactive oxygen species (ROS) production, neutrophil recruitment, and oxidative stress. This leads to hepatocyte necrosis and the release of damage-associated molecular patterns (DAMPs) (15, 16). DAMPs and PAMPs synergistically initiate a “PAMP/DAMP → macrophage → cytokine storm” cascade, perpetuating hepatocellular injury while amplifying and sustaining systemic inflammation (17). (4) Immunoparalysis and SIRS/CARS imbalance: The accumulation of DAMPs/PAMPs in the liver triggers massive cytokine release, shifting hepatocyte death from apoptosis/necrosis to pyroptosis—a highly immunogenic form of cell death. This creates a feed-forward loop where enhanced immunogenicity further increases necrosis risk, ultimately inducing systemic inflammatory response syndrome (SIRS). To counterbalance excessive inflammation, compensatory anti-inflammatory response syndrome (CARS) develops, but this leads to exhaustion and dysfunction of innate/adaptive immune cells (immunoparalysis), elevating infection risks (10, 18–21). These mechanisms collectively explain our findings: ALD-ACLF patients exhibit higher clinical infection rates than HBV-ACLF patients, and once infected, face greater mortality risks. Thus, early infection detection and treatment are critical for this population.

Although the ALD-ACLF group showed higher rates of complications such as ascites and infection compared to the HBV-ACLF group, our study found no statistically significant differences in disease severity (MELD and Child-Pugh scores) or prognosis between the two groups. This suggests that both ALD-ACLF and HBV-ACLF are characterized by rapid disease progression and high mortality rates, consistent with previous studies (6, 9, 22). Notably, the ALD-ACLF group demonstrated significantly lower levels of ALT, AST, and ALB than the HBV-ACLF group. This observation may be attributed to the higher proportion of cirrhotic patients in the ALD-ACLF cohort, aligning with prior research findings (1, 2, 22). While elevated ALT and AST typically reflect the degree of hepatocellular inflammation and necrosis, these enzymes may appear normal or only mildly increased in advanced cirrhosis due to predominant hepatocyte atrophy. Additionally, ethanol consumption depletes hepatic pyridoxal, an essential coenzyme for ALT and AST synthesis, potentially contributing to reduced production of these enzymes (23).

A Japanese multicenter study identified age as a prognostic factor for ACLF patients (24), which shows some discrepancy with our findings. Our study revealed no statistically significant difference in age between deceased and surviving groups in the ALD-ACLF cohort, whereas a significant age difference was observed between corresponding groups in HBV-ACLF patients. This divergence may be attributed to the distinct etiologies of these two ACLF types. For ALD patients progressing to ACLF, alcohol cessation typically occurs, thereby mitigating the primary pathogenic factor. Consequently, their prognosis depends mainly on the extent of liver damage and complications, with age playing a relatively minor role. In contrast, HBV patients often experience increasingly complex clinical conditions with advancing age. Even with standardized antiviral therapy, they remain susceptible to drug resistance and viral reactivation, both of which may negatively impact outcomes. Moreover, elderly HBV-ACLF patients frequently exhibit more severe immune dysfunction and multiple comorbidities. These factors collectively exacerbate disease progression and elevate mortality risk.

Finally, this study found that both TBIL and PT demonstrated AUC values exceeding 0.7 in predicting 90-day mortality risk for both HBV-ACLF and ALD-ACLF patients, indicating good predictive performance. Further multivariate logistic regression analysis confirmed TBIL and PT as independent risk factors for 90-day mortality in both ACLF types, consistent with previous ACLF research findings (1). As a crucial hepatic metabolism product, bilirubin levels directly reflect the liver’s synthetic and excretory functions. During ACLF, impaired liver function leads to bilirubin metabolism disorders and systemic accumulation. Elevated bilirubin levels not only indicate worsening hepatic function but also correlate with potential complications like hepatic encephalopathy, which may further aggravate the condition and worsen prognosis (22). Consequently, bilirubin has been incorporated into various prognostic scoring systems, including the MELD score. Our study similarly observed significantly higher bilirubin levels in deceased patients compared to survivors across both HBV-ACLF and ALD-ACLF groups. Multiple coagulation factors are synthesized in the liver. In ACLF, severe hepatic injury compromises this synthetic capacity. Additionally, the systemic inflammatory response characteristic of ACLF releases cytokines that further suppress hepatic synthetic function, exacerbating coagulation factor deficiency. When complicated by conditions like gastrointestinal bleeding, rapid consumption of coagulation factors may outpace hepatic production, ultimately resulting in prolonged PT.

In addition to TBIL and PT, existing studies have identified WBC as an independent risk factor for mortality in ACLF patients, and this parameter has been incorporated into the CLIF-CACLF score (25, 26). Multiple studies have demonstrated that systemic inflammatory response serves as a hallmark feature of ACLF, with particularly prominent manifestations in ALD-ACLF patients. Notably, our study further revealed that WBC specifically emerged as an independent risk factor for 90-day mortality exclusively in ALD-ACLF patients, while no such correlation was observed in HBV-ACLF cases. This distinction primarily stems from alcohol’s immunosuppressive effects and the consequent inflammatory cascade (27, 28). Mechanistically, alcohol and its metabolites directly damage hepatocytes, triggering the release of damage-associated molecular patterns (DAMPs) and pathogen-associated molecular patterns (PAMPs). These molecules activate immune cells, promoting excessive cytokine release and ultimately inducing systemic inflammatory response (29–32). Furthermore, chronic alcohol consumption compromises immune function, causing leukocyte dysfunction that increases infection risk while exacerbating hepatic injury and failure. In contrast, HBV-ACLF pathogenesis primarily relates to viral replication and localized hepatic immune responses. Although WBC participates in this immune process, its mechanistic role differs fundamentally from alcohol-induced immunopathology, explaining WBC’s more pronounced prognostic value in ALD-ACLF. This differential impact of WBC as a risk factor underscores the distinct pathophysiological mechanisms underlying HBV-ACLF versus ALD-ACLF. An important aspect of our cohort is the absence of liver transplantation, a definitive treatment for ACLF. This circumstance, while highlighting a healthcare resource disparity, uniquely positions our study to elucidate the “natural” disease course and prognosis under maximal medical therapy alone. The risk factors we identified, therefore, are particularly relevant for prognostication in settings where transplant is not readily accessible.

### 4.1 Limitations

While this study yielded significant findings, several limitations should be acknowledged: (1) Limited sample size and single-center design: The small cohort from a single medical institution may not fully represent diverse regional or demographic populations, potentially compromising the statistical accuracy and external validity of the results. (2) Heterogeneity in treatment regimens: Prognostic interpretations may be confounded by therapeutic differences between HBV-ACLF and ALD-ACLF groups. For instance, variations in antiviral therapy for HBV-ACLF or antibiotic use for concurrent infections could influence outcomes. However, these treatment-related data were not systematically collected during the study, precluding further analysis. (3) Static laboratory parameters: The study design did not account for dynamic changes in laboratory indicators throughout the disease course, nor did it stratify patients by infection site or severity. Notably, all enrolled patients were treated at a leading tertiary hospital in the region, reflecting local standards of care. Thus, the findings retain significant regional relevance and provide a reliable reference for identifying high-risk ACLF patients in western Yunnan. We anticipate future multicenter collaborations to validate these results, thereby enhancing their reliability and clinical applicability.

## 5 Conclusion

In summary, patients in the ALD-ACLF group exhibited higher rates of complications such as ascites and infections compared to the HBV-ACLF group, with more severe infectious manifestations. However, the prognosis did not significantly differ between the two ACLF subtypes. TBIL and PT were identified as independent risk factors for 90-day mortality in HBV-ACLF patients, whereas TBIL, PT, and WBC emerged as independent risk factors for 90-day mortality in ALD-ACLF patients. Comprehensive evaluation of these indicators may assist in clinically identifying high-risk populations, thereby optimizing diagnostic and therapeutic decision-making.

## Data Availability

The raw data supporting the conclusions of this article will be made available by the authors, without undue reservation.

## References

[1] Tang X Qi T Li B Li H Huang Z Zhu Z . Tri-typing of hepatitis B-related acute-on-chronic liver failure defined by the world gastroenterology organization. J Gastroenterol Hepatol. (2021) 36:208–16. doi: 10.1111/jgh.15113, 32445263

[2] Mu X Tong J Xu X Chen J Su H Liu X . World gastroenterology organisation classification and a new type-based prognostic model for hepatitis B virus-related acute-on-chronic liver failure. Clin Res Hepatol Gastroenterol. (2021) 45:101548. doi: 10.1016/j.clinre.2020.09.009, 33554865

[3] Batra N Gaidhane SA Kumar S Acharya S. Outcome predictors of acute-on-chronic liver failure: a narrative review. Cureus. (2024) 16:e61655. doi: 10.7759/cureus.61655, 38966452 PMC11223737

[4] Wu T Li J Shao L Xin J Jiang L Zhou Q . Development of diagnostic criteria and a prognostic score for hepatitis B virus-related acute-on-chronic liver failure. Gut. (2018) 67:2181–91. doi: 10.1136/gutjnl-2017-314641, 28928275

[5] Sundaram V Mahmud N Perricone G Katarey D Wong RJ Karvellas CJ . Longterm outcomes of patients undergoing liver transplantation for acute-on-chronic liver failure. Liver Transpl. (2020) 26:1594–602. doi: 10.1002/lt.25831, 32574423

[6] Duan XZ Liu FF Tong JJ Yang HZ Chen J Liu XY . Granulocyte-colony stimulating factor therapy improves survival in patients with hepatitis B virus-associated acute-on-chronic liver failure. World J Gastroenterol. (2013) 19:1104–10. doi: 10.3748/wjg.v19.i7.1104, 23467275 PMC3581999

[7] Gustot T Jalan R. Acute-on-chronic liver failure in patients with alcohol-related liver disease. J Hepatol. (2019) 70:319–27. doi: 10.1016/j.jhep.2018.12.008, 30658733

[8] Moreau R Jalan R Gines P Pavesi M Angeli P Cordoba J . Acute-on-chronic liver failure is a distinct syndrome that develops in patients with acute decompensation of cirrhosis. Gastroenterology. (2013) 144:1426–1437.e9. doi: 10.1053/j.gastro.2013.02.042 23474284

[9] Weng WZ Chen JF Peng XH Weng W Chen J Peng X . Risk factors for underlying comorbidities and complications in patients with hepatitis B virus-related acute-on-chronic liver failure. Epidemiol Infect. (2022) 150:e147. doi: 10.1017/s0950268822001169, 35788251 PMC9354478

[10] Shang DB Xiang XG. Pathogenesis and therapeutic advances in acute-on-chronic liver failure. J Clin Hepatol. (2021) 37:765–9. doi: 10.3969/j.issn.1001-5256.2021.04.005

[11] Zhang B Humar Z Zhang SY Zhu B Huang HM Tian J . Advances in pathogenesis and treatment of hepatitis B virus-related acute-on-chronic liver failure. Chin J Liver Dis. (2023) 15:28–33. doi: 10.3969/j.issn.1674-7380.2023.01.005

[12] Wang L Fouts DE Stärkel P Hartmann P Chen P Llorente C . Intestinal REG3 lectins protect against alcoholic steatohepatitis by reducing mucosa-associated microbiota and preventing bacterial translocation. Cell Host Microbe. (2016) 19:227–39. doi: 10.1016/j.chom.2016.01.003, 26867181 PMC4786170

[13] Albillos A de Gottardi A Rescigno M. The gut-liver axis in liver disease: pathophysiological basis for therapy. J Hepatol. (2020) 72:558–77. doi: 10.1016/j.jhep.2019.10.003, 31622696

[14] Trebicka J Bork P Krag A Arumugam M. Utilizing the gut microbiome in decompensated cirrhosis and acute-on-chronic liver failure. Nat Rev Gastroenterol Hepatol. (2021) 18:167–80. doi: 10.1038/s41575-020-00376-3, 33257833

[15] Jophlin L Singal AK. Liver biopsy in patients with alcohol-associated liver disease with acute-on-chronic liver failure. J Clin Exp Hepatol. (2022) 12:544–50. doi: 10.1016/j.jceh.2021.08.009, 35535109 PMC9077173

[16] Philips CA Augustine P Yerol PK Rajesh S Mahadevan P. Severe alcoholic hepatitis: current perspectives. Hepat Med. (2019) 11:97–108. doi: 10.2147/HMER.S197933, 31496843 PMC6691395

[17] Avila MA Dufour JF Gerbes AL Zoulim F Bataller R Burra P . Recent advances in alcohol-related liver disease (ALD): summary of a gut round table meeting. Gut. (2020) 69:764–80. doi: 10.1136/gutjnl-2019-319720, 31879281 PMC7236084

[18] Sendler M van den Brandt C Glaubitz J Wilden A Golchert J Weiss FU . NLRP3 inflammasome regulates development of systemic inflammatory response and compensatory anti-inflammatory response syndromes in mice with acute pancreatitis. Gastroenterology. (2020) 158:253–269.e14. doi: 10.1053/j.gastro.2019.09.040, 31593700

[19] Martin-Mateos R Alvarez-Mon M Albillos A. Dysfunctional immune response in acute-on-chronic liver failure: it takes two to tango. Front Immunol. (2019) 10:973. doi: 10.3389/fimmu.2019.00973 31118937 PMC6504833

[20] Min Y Tong J Wang BY. Acute-on-chronic liver failure associated with alcoholic hepatitis. Chin J Front Med Sci. (2023) 15:12–7. doi: 10.12037/YXQY.2023.02-02

[21] Sun J Guo H Yu X Chen J Zhu H Qi X . Evaluation of prognostic value of neutrophil-to-lymphocyte ratio in patients with acute-on-chronic liver failure or severe liver injury from chronic HBV infection. Eur J Gastroenterol Hepatol. (2021) 33:e670–80. doi: 10.1097/MEG.0000000000002207, 34074984

[22] Yu Z Zhang Y Cao Y Xu M You S Chen Y . A dynamic prediction model for prognosis of acute-on-chronic liver failure based on the trend of clinical indicators. Sci Rep. (2021) 11:1810. doi: 10.1038/s41598-021-81431-0, 33469110 PMC7815739

[23] Ye MC Li Y Xiao L Yang XZ Geng AW Shen ML . Clinical characteristics of 155 patients with alcoholic liver disease. J Pract Hepatol. (2017) 20:60–4. doi: 10.3969/j.issn.1672-5069.2017.01.016

[24] Nakayama N Uemura H Uchida Y Tomiya T Ido A Inoue K . A multicenter pilot survey to clarify the clinical features of patients with acute-on-chronic liver failure in Japan. Hepatol Res. (2018) 48:303–12. doi: 10.1111/hepr.13064, 29341357

[25] Bernsmeier C Cavazza A Fatourou EM Theocharidou E Akintimehin A Baumgartner B . Leucocyte ratios are biomarkers of mortality in patients with acute decompensation of cirrhosis and acute-on-chronic liver failure. Aliment Pharmacol Ther. (2020) 52:855–65. doi: 10.1111/apt.15932, 32683724

[26] Li J Liang X You S Feng T Zhou X Zhu B . Development and validation of a new prognostic score for hepatitis B virus-related acute-on-chronic liver failure. J Hepatol. (2021) 75:1104–15. doi: 10.1016/j.jhep.2021.05.026, 34090929

[27] Hernaez R Solà E Moreau R Ginès P. Acute-on-chronic liver failure: an update. Gut. (2017) 66:541–53. doi: 10.1136/gutjnl-2016-312670, 28053053 PMC5534763

[28] Bernardi M Moreau R Angeli P Schnabl B Arroyo V. Mechanisms of decompensation and organ failure in cirrhosis: from peripheral arterial vasodilation to systemic inflammation hypothesis. J Hepatol. (2015) 63:1272–84. doi: 10.1016/j.jhep.2015.07.004, 26192220

[29] Moreau R. The pathogenesis of ACLF: the inflammatory response and immune function. Semin Liver Dis. (2016) 36:133–40. doi: 10.1055/s-0036-1583199, 27172355

[30] Takeuchi O Akira S. Pattern recognition receptors and inflammation. Cell. (2010) 140:805–20. doi: 10.1016/j.cell.2010.01.022, 20303872

[31] Kono H Rock KL. How dying cells alert the immune system to danger. Nat Rev Immunol. (2008) 8:279–89. doi: 10.1038/nri2215, 18340345 PMC2763408

[32] Iwasaki A Medzhitov R. Control of adaptive immunity by the innate immune system. Nat Immunol. (2015) 16:343–53. doi: 10.1038/ni.3123, 25789684 PMC4507498

